# Community awareness, symptom recognition, and perceived causes of skin neglected tropical diseases in selected regions of Ghana

**DOI:** 10.3389/fpubh.2026.1904661

**Published:** 2026-09-22

**Authors:** Eric Koka, Selorm Adinkra, Abigail Abban, Samuel Antwi, Hannah Benedicta Taylor-Abdulai, Quao Benedict Okoe

**Affiliations:** 1 Department of Sociology and Anthropology, University of Cape Coast, Cape Coast, Ghana; 2 School of Public Health, University of Ghana, Accra, Ghana; 3 School of Medical Sciences, University of Cape Coast, Cape Coast, Ghana; 4 Ankaful Leprosy/General Hospital, Ghana Health Service, Cape Coast, Ghana

**Keywords:** Buruli ulcer, leprosy, skin neglected tropical diseases (skin NTDs), stigma, yaws

## Abstract

**Background:**

Leprosy and other skin neglected tropical diseases, including Buruli ulcer and yaws, remain significant public health challenges in Ghana. Despite control efforts, these conditions continue to cause disability, stigma, and social exclusion, particularly in rural and underserved communities. Misconceptions rooted in cultural and spiritual beliefs further delay care-seeking and reinforce discrimination.

**Objective:**

This study assessed community awareness, symptom recognition, and perceived causes of leprosy and other skin NTDs across four regions of Ghana, and examined how these varied by socio-demographic characteristics, to inform culturally appropriate interventions for early detection and stigma reduction.

**Methods:**

An analytical cross-sectional survey was conducted among 1,067 adults in Greater Accra, Eastern, Bono, and Savannah Regions of Ghana. Data were collected using structured questionnaires administered via Kobo Collect. Descriptive statistics summarized socio-demographic characteristics and outcome variables, including awareness, symptom recognition, and perceived causes of skin neglected tropical diseases. Associations between these outcomes and socio-demographic factors were assessed using chi-square tests, with statistical significance set at *p* ≤ 0.05.

**Results:**

Overall awareness of skin NTDs was high (75.6%) and was significantly associated with respondents’ educational level (*χ*^2^ = 38.081, *p* < 0.001) and region of residence (*χ*^2^ = 42.661, *p* < 0.001). Respondents with higher educational attainment and those from the Bono Region showed higher levels of awareness, whereas awareness was lowest among respondents from the Eastern Region. Symptom recognition was also assessed, with wounds (32.6%) and skin lesions (21.7%) being the most commonly identified manifestations. Perceived causes reflected a combination of biomedical and sociocultural explanations; bacterial infection and genetic factors were the most frequently reported causes, although supernatural beliefs remained prevalent, particularly among respondents with lower educational attainment.

**Conclusion:**

This study shows that community awareness, symptom recognition, and perceived causes of skin NTDs in Ghana differ across socio-demographic and regional contexts. Interventions should prioritize communities and population groups with lower awareness, strengthen education on early skin NTD symptoms and their biomedical causes, and engage local leaders to address misconceptions and stigma, thereby improving early detection and timely healthcare-seeking.

## Introduction

Leprosy and other neglected tropical diseases (NTDs), including Buruli ulcer and yaws, remain persistent global public health challenges despite sustained control and elimination efforts. Globally, NTDs affect over 1.7 billion people, predominantly in low- and middle-income countries, where they contribute to chronic disability, disfigurement, reduced productivity, and long-term social exclusion (1). Leprosy continues to record over 200,000 new cases annually worldwide, with India, Brazil, and Indonesia accounting for a significant proportion of cases. Buruli ulcer has been reported in more than 30 countries, particularly in West and Central Africa, while yaws, although targeted for eradication, continues to re-emerge in several tropical settings. These diseases are strongly associated with poverty-related conditions such as inadequate sanitation, overcrowding, limited access to healthcare services, and low health literacy, reflecting persistent global health inequities (2).

In Africa, the burden of skin NTDs remains substantial, with West and Central Africa serving as major endemic zones. Countries such as Ghana, Nigeria, Côte d’Ivoire, and the Democratic Republic of Congo continue to report cases of Buruli ulcer, yaws, and leprosy despite ongoing control interventions (3). Ghana, for example, has historically been one of the highest reporting countries for Buruli ulcer globally, while yaws has re-emerged in several rural districts after periods of apparent decline. Leprosy, though significantly reduced, remains endemic in selected districts across the continent (4). Weak surveillance systems, delayed diagnosis, and limited access to early treatment continue to sustain transmission. In addition, reliance on traditional healing systems and socio-cultural interpretations of disease contributes to delayed care-seeking and ongoing disability (5, 6).

In Ghana, skin NTDs exhibit marked geographical and socio-economic variation. Buruli ulcer is predominantly found in swampy and riverine communities, particularly in parts of the Ashanti, Central, and Eastern regions, where environmental conditions favor transmission. Yaws has been reported in rural and hard-to-reach communities in both northern and southern belts, where access to healthcare services remains limited (3, 7). Leprosy, although substantially reduced due to national control efforts, continues to present sporadic cases across regions, including Greater Accra, Eastern, Bono, and Savannah (8, 9). Ghana Health Service surveillance reports indicate that new and relapsed cases of skin NTDs are still recorded annually, suggesting persistent gaps in early detection, awareness, and community-level prevention.

Beyond their biomedical burden, these diseases are strongly associated with stigma, discrimination, and social exclusion. In Ghana, leprosy is widely misperceived as hereditary, incurable, or a punishment for wrongdoing (8, 10). Such beliefs foster fear-driven avoidance, leading to exclusion from marriage, employment, and social participation (11). Similar misconceptions exist for Buruli ulcer and yaws, where illness is often attributed to witchcraft, spiritual forces, or moral failure (12, 13). These perceptions delay timely health-seeking and reinforce dependence on traditional medicine, often worsening disease outcomes.

The consequences are far-reaching, affecting individuals, families, and communities. Affected persons frequently experience physical disability alongside psychological distress, such as anxiety, depression, and reduced self-esteem, due to visible deformities and social rejection (14, 15). Families also bear significant economic burdens through caregiving responsibilities, reduced productivity, and increased healthcare costs (9, 16). These effects reinforce cycles of poverty and inequality, limiting inclusive development.

Given these challenges, strengthening community awareness and understanding of skin NTDs is essential for achieving SDG 3, particularly targets on ending neglected tropical diseases (SDG 3.3) and achieving universal health coverage (SDG 3.8). It also supports broader development priorities related to reducing inequalities (SDG 10) and ending poverty (SDG 1), given the strong bidirectional relationship between NTDs and socio-economic deprivation. However, evidence in Ghana remains largely fragmented and geographically limited, with few studies providing comparative insights across multiple ecological zones (17, 18). This study, therefore, examines community perceptions and awareness toward persons with leprosy and other skin NTDs across four regions of Ghana, focusing on awareness, symptom recognition, and perceived causes, to inform culturally appropriate interventions that reduce stigma, improve early detection, and promote social inclusion.

In Ghana, community-level research on skin neglected tropical diseases (NTDs) has increased over the past decade but remains uneven, with much of the available evidence concentrated on Buruli ulcer in selected endemic communities. Studies conducted in Ga West, Ga South, and Obom have examined community knowledge, attitudes, practices, health-seeking behaviors, and cultural interpretations of wounds and Buruli ulcer, demonstrating that local beliefs and social experiences influence disease recognition, treatment decisions, and prevention practices (5, 6, 12, 13, 17, 18).

Leprosy-related research in Ghana has primarily focused on stigma, discrimination, and the lived experiences of affected individuals, highlighting the social consequences associated with visible skin conditions (9). More recent studies have expanded attention to stigma experiences, coping mechanisms, and community responses among individuals affected by Buruli ulcer and yaws, while also examining the role and capacity needs of community-based surveillance volunteers in integrated skin NTD control (2, 15, 19). Epidemiological investigations have further contributed evidence on the distribution, geographical patterns, and risk factors of Buruli ulcer and yaws in Ghana (3, 4, 7).

Despite these contributions, important gaps remain. Most existing community-level studies have focused on single diseases, particularly Buruli ulcer, and have been conducted within specific endemic localities. Consequently, limited evidence exists on how awareness, symptom recognition, and perceived causes of different skin NTDs compare across Ghana’s diverse ecological zones. This limitation is important because the World Health Organization promotes an integrated approach to skin NTD control as part of efforts to achieve the Sustainable Development Goals, including reducing the burden of neglected diseases and improving access to essential health services (1).

Furthermore, few studies have examined how community understanding of skin NTDs varies simultaneously according to socio-demographic characteristics such as education, gender, and geographical location. Such evidence is essential for designing targeted health education interventions, improving early case detection, strengthening community-based surveillance, and addressing stigma associated with visible skin diseases.

This study therefore assessed community awareness, symptom recognition, and perceived causes of leprosy and other skin NTDs across four regions of Ghana and examined variations by education, gender, and region. Since skin NTDs often present with visible and potentially disfiguring lesions that carry significant social and cultural meanings, community perceptions influence whether early signs are recognized, whether affected individuals seek appropriate biomedical care, and how they are treated within their communities. Understanding these factors is therefore critical for developing culturally appropriate interventions that improve early detection, promote timely treatment, and reduce stigma in alignment with SDG 3.3 and SDG 3.8.

## Methodology

### Study design

The study employed a quantitative analytical cross-sectional survey design to assess community awareness, symptom recognition, and perceived causes of leprosy and skin neglected tropical diseases in selected regions of Ghana. This design was appropriate for examining the distribution of these outcomes and their associations with socio-demographic characteristics at a single point in time across diverse populations and geographical settings.

It further allowed for the examination of differences in responses across socio-demographic characteristics and regions, thereby enabling comparison of awareness levels, symptom recognition patterns, and causal beliefs related to skin NTDs within the study population.

### Study area

The study was conducted in four purposively selected regions of Ghana: Greater Accra, Eastern, Bono, and Savannah. These regions were selected to reflect Ghana’s geographic, socio-economic, and ecological diversity, encompassing urban, peri-urban, and rural settings.

The Greater Accra Region, the most urbanized and densely populated, represented highly urban settings with relatively better access to health services. The Eastern Region included both urban and rural communities and reflected a mixed socio-economic and agricultural setting. The Bono Region lies in the transitional forest–savannah zone and provided insight into semi-urban and agrarian livelihoods influenced by cultural beliefs. The Savannah Region, predominantly rural with dispersed settlements, represented communities where traditional health beliefs and limited access to healthcare are more pronounced (5, 9).

Within the Greater Accra Region, data were collected from selected urban and peri-urban communities within the Accra Metropolitan Area and adjoining settlements. These included densely populated neighborhoods where formal employment and higher education levels were more common, as well as peri-urban settlements with mixed socio-economic characteristics.

### Study population

The target population comprised adults aged 18 years and above residing in selected communities within the Greater Accra, Eastern, Bono, and Savannah Regions of Ghana during the study period. These regions were selected to capture variations in urban, peri-urban, and rural settings. A total of 1,067 participants were included in the study. Eligibility criteria included being a resident of the selected communities, aged 18 years or older, and providing informed consent to participate. Only individuals who met the eligibility criteria and were available during the data collection period were administered the structured questionnaire.

### Sample size estimation

The sample size for the study was determined using Cochran’s formula for estimating sample size for proportions:

$$n=\frac{Z^{2}p(1-p)}{e^{2}}$$

Where: n = required sample size.

Z = standard normal deviate corresponding to the desired confidence level (1.96 for 95% confidence level).

p = estimated proportion of 0.33, adopted as an anticipated proportion for sample size estimation due to the absence of a single national estimate for community awareness and symptom recognition of skin neglected tropical diseases in Ghana, and.

e = margin of error (0.0283).

$$n=\frac{{\left(1.96\right)}^{2}0.33(1-0.33)}{{\left(0.0283\right)}^{2}}$$

n=1060.54

Substituting these values into Cochran’s formula yielded an estimated sample size of approximately 1,060 participants. To compensate for potential non-response and incomplete questionnaires, the sample size was slightly adjusted upward, resulting in a final sample size of 1,067 respondents.

An anticipated proportion of (*p* = 0.33) was specified *a priori* as a pragmatic planning assumption representing a one-third outcome scenario during the planning of the survey. This value was used for sample-size determination and was not intended to represent an established national prevalence of community awareness or symptom recognition of skin NTDs. Using a 95% confidence level (*Z* = 1.96) and a margin of error of 0.0283 produced a minimum estimated sample size of approximately 1,061 participants. The final achieved sample was 1,067 respondents. This achieved sample is also approximately equivalent to the sample required under the maximum-variability assumption of (*p* = 0.50), with a 3% margin of error at the 95% confidence level.

### Sampling procedure

A multi-stage sampling technique was employed to ensure adequate representation across regions, districts, communities, households, and individuals. First, the four regions were purposively selected based on geographical variation and the presence of diverse urban, peri-urban, and rural settings. Second, districts within each selected region were obtained from the respective Regional Coordinating Councils and selected using a simple random sampling approach. Third, selected districts were stratified into communities, from which urban and rural communities were selected through random sampling to ensure contextual diversity.

Fourth, household lists were compiled with the support of community leaders and local guides. Systematic sampling was then applied to select households using a predetermined sampling interval based on the estimated number of households and the allocated sample size for each community. Finally, all eligible adults within selected households were identified. Where more than one eligible participant was available in a household, one participant was selected using a computer-generated random number procedure to ensure equal probability of selection and minimize selection bias.

### Study variables and measurement

The main outcome variables included awareness of skin NTDs, symptom recognition, and perceived causes of skin NTDs. Independent variables included socio-demographic characteristics such as age, gender, education, occupation, marital status, and region of residence. Awareness was measured as a binary variable (Yes/No). Symptom recognition and perceived causes were measured using multiple-response categorical items, where respondents selected the option they most strongly associated with skin NTDs.

### Data collection procedures

Data were collected between 4th and 24th September 2024 using a structured questionnaire administered electronically via the Kobo Collect application. The tool captured data on socio-demographic characteristics, awareness of skin NTDs, symptom recognition, and perceived causes of the diseases.

Data collection was conducted in two phases. The first phase covered the Savannah and Bono Regions, while the second phase covered the Greater Accra and Eastern Regions. In rural communities, interviews were conducted mainly in the evenings due to daytime occupational activities. In urban areas such as Greater Accra, participant verification and trust-building were necessary due to higher levels of scrutiny, while in communities such as Adoagyiri in the Eastern Region, respondents showed high willingness to participate after brief explanations.

### Data access and management

Completed questionnaires were automatically stored in the KoboToolbox system and downloaded for cleaning and analysis. Data were checked for completeness, consistency, and duplication before analysis. All datasets were anonymized using unique identifiers to ensure confidentiality. Access to raw data was restricted to the research team only.

### Data processing and analysis

Data were downloaded from Kobo Collect in Excel format, cleaned, and exported into SPSS Statistical software for analysis. Cleaning involved checking for missing values, duplicates, inconsistencies, and outliers. Incomplete responses were excluded based on predefined criteria. Variables were coded and categorized for analysis.

Descriptive statistics, including frequencies and percentages, were used to summarize socio-demographic characteristics and key study variables such as awareness, symptom recognition, and perceived causes of skin NTDs. These provided an overview of response patterns across participants. Cross-tabulations were conducted to examine relationships between socio-demographic factors (age, gender, education, occupation, and region) and outcome variables. This allowed comparison of distributions across subgroups.

Chi-square tests were used to assess associations between categorical variables. Statistical significance was set at *p* ≤ 0.05. Significant results indicated meaningful relationships between variables rather than chance variation. Findings were presented using tables and figures, combining descriptive and inferential outputs to support the interpretation of patterns and associations in the data.

### Ethical considerations and consent

Ethical approval for the study was obtained from the Ghana Health Service Ethics Review Committee (GHS-ERC: 005/04/24). All participants provided informed consent before participation after being informed about the purpose of the study, procedures involved, potential risks, and their right to withdraw at any time without penalty. Confidentiality and anonymity were strictly maintained through the use of coded identifiers instead of personal information. Data were stored securely and used solely for academic purposes.

## Results

### Socio-demographic characteristics of respondents

Table 1 presents the socio-demographic characteristics of the respondents. In terms of age distribution, the largest proportion of respondents was within the 18–27 years age group (39.4%), followed by 28–37 years (32.3%). Relatively smaller proportions were observed among those aged 38–47 years (16.7%), 48–57 years (6.9%), 58–67 years (3.3%), 68–77 years (0.9%), and 78–88 years (0.4%).

**Table 1:** Socio-demographic characteristics of respondents

Variable	Frequency (N)	Percent (%)
Age		
18–27	420	39.4
28–37	345	32.3
38–47	178	16.7
48–57	74	6.9
58–67	35	3.3
68–77	10	0.9
78–88	5	0.5
Sex		
Female	470	44.0
Male	597	56.0
Marital Status		
Cohabiting	20	1.9
Divorced	19	1.8
Married	439	41.1
Separated	11	1.0
Single	564	52.9
Widowed	14	1.3
Educational Qualification		
JHS	313	29.3
No formal education	108	10.1
Primary education	151	14.2
SHS	365	34.2
Tertiary	112	10.5
VOC/TECH	18	1.7
Occupation		
Casual labor	121	11.3
Farming	73	6.8
Not employed	104	9.7
Official employee	58	5.4
Student	104	9.7
Trade/Business	607	56.9
Religion		
Christianity	948	88.8
Islam	110	10.3
Other	5	0.5
Traditional African religions	4	0.4

Source: Author’s construct, 2025.

Regarding sex, the majority of respondents were male (56.0%), while females constituted 44.0% of the sample. For marital status, more than half of the respondents were single (52.9%), followed by those who were married (41.1%). Smaller proportions were cohabiting (1.9%), divorced (1.8%), separated (1.0%), and widowed (1.3%).

In terms of educational attainment, most respondents had secondary-level education, with 34.2% having Senior High School (SHS) and 29.3% having Junior High School (JHS). A further 14.2% had primary education, while 10.5% had tertiary education. Respondents with no formal education accounted for 10.1%, and those with vocational/technical education made up 1.7%.

With respect to occupation, the majority of respondents were engaged in trade or business activities (56.9%). This was followed by casual labor (11.3%), unemployed persons (9.7%), students (9.7%), farming (6.8%), and formal employment (5.4%). Finally, the religious composition of respondents showed that most were Christians (88.8%), followed by Muslims (10.3%), while a very small proportion practiced other religions (0.5%) or traditional African religions (0.4%).

Table 2 presents the cross-tabulation between selected socio-demographic characteristics and awareness of skin neglected tropical diseases (skin NTDs) among respondents. Overall, 807 of the 1,067 respondents (75.6%) reported that they had heard about skin NTDs, while 260 (24.4%) indicated otherwise.

**Table 2:** Cross-tabulation of socio-demographic characteristics and awareness of skin NTDs among respondents.

Variable	Category	No *n* (%)	Yes *n* (%)	Total (N)	% Yes	Pearson Chi-Square	*p*-value
Educational Level	No formal education	123 (33.7)	242 (66.3)	365	66.3	38.081	<0.001
	Primary education	5 (27.8)	13 (72.2)	18	72.2		
	JHS	31 (27.7)	81 (72.3)	112	72.3		
	SHS	66 (21.1)	247 (78.9)	313	78.9		
	VOC/TECH	19 (17.6)	89 (82.4)	108	82.4		
	Tertiary	16 (10.6)	135 (89.4)	151	89.4		
	Total	260 (24.4)	807 (75.6)	1,067	75.6		
Sex	Female	111 (23.6)	359 (76.4)	470	76.4	0.257	0.612
	Male	149 (25.0)	448 (75.0)	597	75		
	Total	260 (24.4)	807 (75.6)	1,067	75.6		
Region	Bono	7 (5.6)	119 (94.4)	126	94.4	42.661	<0.001
	Eastern	105 (34.4)	200 (65.6)	305	65.6		
	Greater Accra	136 (23.9)	432 (76.1)	568	76.1		
	Savannah	12 (17.6)	56 (82.4)	68	82.4		
	Total	260 (24.4)	807 (75.6)	1,067	75.6		

Source: Author’s construct, 2025.

Awareness of skin NTDs varied significantly across educational levels (*χ*^2^ = 38.081, *p* < 0.001). Respondents with tertiary education recorded the highest awareness level (89.4%), followed by those with vocational/technical education (82.4%) and SHS education (78.9%). Awareness was comparatively lower among respondents with JHS education (72.3%) and primary education (72.2%), while those with no formal education recorded 66.3%. This indicates that higher educational attainment was generally associated with increased awareness of skin NTDs.

Regarding sex, awareness levels were similar between males and females. Among female respondents, 76.4% reported awareness of skin NTDs compared with 75.0% among males. The observed difference was not statistically significant (*χ*^2^ = 0.257, *p* = 0.612), suggesting that awareness did not differ meaningfully by gender in the study population.

Regional variations in awareness were more pronounced and statistically significant (*χ*^2^ = 42.661, *p* < 0.001). The Bono Region recorded the highest level of awareness (94.4%), followed by the Savannah Region (82.4%) and the Greater Accra Region (76.1%). The Eastern Region recorded the lowest awareness level (65.6%). These findings indicate substantial geographical disparities in awareness of skin NTDs across the study areas, with higher awareness generally observed in some regions compared to others.

Table 3 presents the association between socio-demographic characteristics and respondents’ recognition of symptoms associated with skin neglected tropical diseases (NTDs). Respondents identified the symptom they most strongly associated with skin NTDs, with wounds (348) and skin lesions (232) being the most frequently recognized symptoms overall, while nodules (62) and patches (72) were less commonly identified.

**Table 3:** Cross-tabulation of socio-demographic characteristics and symptom recognition of skin NTDs among respondents.

Variable	Category	Muscle weakness	Nodules	Not sure	Numbness in extremities	Patches	Skin Lesions	Wounds	Total	Pearson Chi-Square	*p*-value
Educational Level	JHS	17	19	47	45	13	71	101	313	59.205	0.001
	No formal education	6	3	11	14	17	26	31	108		
	Primary education	19	4	20	15	12	34	47	151		
	SHS	25	29	38	50	23	69	131	365		
	Tertiary	8	6	8	21	7	24	38	112		
	VOC/TECH	1	1	3	5	0	8	0	18		
	Total	76	62	127	150	72	232	348	1,067		
Sex	Female	34	26	63	67	43	105	132	470	14.341	0.026
	Male	42	36	64	83	29	127	216	597		
	Total	76	62	127	150	72	232	348	1,067		
Region	Bono	9	0	4	6	18	33	32	102	112.29	0.001
	Eastern	8	10	8	9	2	21	44	102		
	Greater Accra	7	6	17	19	5	19	26	99		
	Savannah	0	1	1	15	19	25	38	99		

Source: Author’s construct, 2025.

With respect to educational level, respondents with SHS education most frequently identified wounds (131) and skin lesions (69), while respondents with JHS education also commonly recognized wounds (101) and skin lesions (71). Participants with no formal education predominantly identified wounds (31) and skin lesions (26). The association between educational level and symptom recognition was statistically significant (*χ*^2^ = 59.205, *p* = 0.001), indicating that recognition of skin NTD symptoms varied significantly across educational categories.

Sex differences in symptom recognition were also observed. Among females, wounds (132) and skin lesions (105) were the most commonly recognized symptoms, whereas males similarly identified wounds (216) and skin lesions (127) more frequently than other symptoms. A statistically significant association was found between gender and symptom recognition (*χ*^2^ = 14.341, *p* = 0.026), suggesting that males and females differed in the symptoms they most associated with skin NTDs.

Regional variations in symptom recognition were equally evident. Respondents from the Bono Region commonly identified skin lesions (33) and wounds (32), while participants from the Eastern Region most frequently identified wounds (44). In the Savannah Region, wounds (38), skin lesions (25), and patches (19) were the dominant responses. A statistically significant association existed between region and symptom recognition (*χ*^2^ = 112.29, *p* = 0.001), indicating that knowledge of skin NTD symptoms differed significantly across regions.

Table 4 presents the cross-tabulation of respondents’ educational level and their perceived causes of skin neglected tropical diseases (NTDs). Overall, genetic factors and bacterial infection were the most frequently reported perceived causes across most educational groups, while “other” causes were the least reported.

**Table 4:** Cross-tabulation of educational level and perceived causes of skin NTDs.

Educational level	Bacterial infection	Genetic factors	Not sure	Other	Poor hygiene	Supernatural causes
JHS	67	106	41	8	37	54
No formal education	30	36	19	1	7	15
Primary education	33	58	22	4	16	18
SHS	121	111	38	4	51	40
Tertiary	30	37	19	0	14	12
VOC/TECH	4	8	3	0	1	2

Source: Author’s construct, 2025.

Among respondents with JHS education, genetic factors (106) were the most frequently reported cause, followed by bacterial infection (67) and supernatural causes (54). Respondents with SHS education similarly reported bacterial infection (121) and genetic factors (111) most frequently, followed by poor hygiene (51) and supernatural causes (40). Those with tertiary education identified genetic factors (37) and bacterial infection (30) as the leading perceived causes, while poor hygiene (14) and supernatural causes (12) were less frequently reported.

Among respondents with primary education, genetic factors (58) and bacterial infection (33) were the most commonly reported causes, followed by supernatural causes (18) and poor hygiene (16). Respondents with no formal education also predominantly identified genetic factors (36) and bacterial infection (30), although some reported uncertainty (19) and supernatural causes (15). Similarly, respondents with vocational/technical education mainly identified genetic factors (8) and bacterial infection (4), with relatively few reporting other perceived causes. Overall, genetic factors and bacterial infection were consistently prominent perceived causes across educational levels, although perceptions varied regarding supernatural causes, poor hygiene, and uncertainty.

Table 5 presents the distribution of respondents’ perceived causes of skin neglected tropical diseases (NTDs) according to gender and region. Across both males and females, genetic factors and bacterial infection were the most frequently reported perceived causes. Among male respondents, 218 identified genetic factors and 161 identified bacterial infection, while among female respondents, 138 attributed skin NTDs to genetic factors and 124 to bacterial infection. Similar proportions of males and females also attributed skin NTDs to supernatural causes and poor hygiene. In addition, 75 males and 67 females reported being unsure of the causes. The chi-square test showed no statistically significant association between gender and perceived causes of skin NTDs (*χ*^2^ = 8.963, *p* = 0.111), indicating that perceptions of causation were generally similar between male and female respondents.

**Table 5:** Cross-tabulation of gender, region, and perceived causes of skin NTDs among respondents.

Variable	Category	Bacterial infection	Genetic factors	Not sure	Other	Poor hygiene	Supernatural causes	Total	Pearson Chi-Square	*p*-value
Gender	Female	124	138	67	7	65	69	470	8.963	0.111
	Male	161	218	75	10	61	72	597		
	Total	285	356	142	17	126	141	1,067		
Region	Bono	30	44	16	6	6	24	126	47.284	<0.001
	Eastern	92	106	42	1	48	16	305		
	Greater Accra	148	186	74	9	65	86	568		
	Savannah	15	20	10	1	7	15	68		
	Total	285	356	142	17	126	141	1,067		

Source: Author’s construct, 2025.

Regional differences in perceived causes were, however, statistically significant (*χ*^2^ = 47.284, *p* < 0.001). In the Greater Accra Region, genetic factors (186) and bacterial infection (148) were the most frequently reported causes, although supernatural causes were also relatively common (86). Respondents from the Eastern Region similarly identified genetic factors (106) and bacterial infection (92) as the leading causes, with a notable proportion attributing skin NTDs to poor hygiene (48). In the Bono Region, respondents frequently associated skin NTDs with genetic factors (44), bacterial infection (30), and supernatural causes (24). In the Savannah Region, genetic factors (20), bacterial infection (15), and supernatural causes (15) were the dominant responses. Overall, the findings indicate that perceptions regarding the causes of skin NTDs varied significantly across regions, reflecting possible cultural, environmental, and contextual differences in understanding skin NTDs.

## Discussion

The findings of this study provide important insights into community awareness, symptom recognition, and perceived causes of leprosy and other neglected tropical diseases (NTDs) in Ghana. The main finding was that although awareness of skin NTDs was generally high among respondents, significant differences existed in symptom recognition and perceived causes across educational levels and regions. Higher educational attainment was associated with better awareness and greater acceptance of biomedical explanations, while misconceptions relating to supernatural causes and poor hygiene remained more common among less educated respondents and in some regions. Gender differences in overall awareness were minimal, although males and females differed in the symptoms they most commonly recognized. These findings indicate that community understanding of skin NTDs in Ghana remains uneven and strongly influenced by socio-demographic and geographical factors.

Overall, more than three-quarters of respondents reported that they had heard about skin NTDs, suggesting that awareness of these diseases exists within many communities. However, awareness did not necessarily translate into an accurate understanding of symptoms and disease causation. One of the major findings of the study was the significant association between educational level and awareness of skin NTDs. Respondents with tertiary education demonstrated the highest awareness levels, whereas those with no formal education recorded the lowest awareness. This finding is consistent with studies conducted in Ghana and elsewhere, which have shown that educational attainment improves health literacy and increases exposure to public health information (19, 20). Similarly, Renzaho et al. (12) reported that individuals with higher educational backgrounds in Ghana were more likely to possess accurate knowledge about Buruli ulcer transmission and treatment. The higher awareness among educated respondents in the present study may be explained by greater access to formal information channels such as schools, media platforms, and healthcare services. Education may also improve individuals’ ability to interpret disease-related information and distinguish biomedical explanations from cultural misconceptions. Nevertheless, the finding that awareness was not absent among less-educated groups suggests that community sensitization activities and prior exposure to skin NTD cases may have contributed to some level of general awareness within communities.

The study also found significant regional differences in awareness and perceptions of skin NTDs. Respondents from the Bono Region recorded the highest levels of awareness, while those from the Eastern Region reported comparatively lower levels. Regional variations were similarly observed in symptom recognition and perceived causes of disease. These findings are consistent with earlier Ghanaian studies that demonstrated geographical differences in community knowledge and health-seeking behaviors regarding Buruli ulcer and other skin NTDs (15, 18). The observed differences may reflect variations in disease endemicity, intensity of public health interventions, and accessibility of healthcare services across regions. Areas that have experienced repeated outbreaks or targeted health campaigns are more likely to have populations with higher awareness and familiarity with disease symptoms. Koka et al. (6) similarly observed that prolonged exposure to health education activities in endemic communities improved recognition of Buruli ulcer symptoms and encouraged early reporting. In contrast, regions with limited health promotion activities or weaker surveillance systems may continue to experience lower levels of awareness and persistent misconceptions regarding skin NTDs.

Another important finding relates to symptom recognition. Wounds and skin lesions were the most commonly recognized symptoms across all socio-demographic groups, whereas nodules and patches were less frequently identified. Educational level, sex, and region all showed significant associations with symptom recognition. These findings are broadly consistent with previous studies in Ghana and Cameroon, where visible and advanced manifestations of skin NTDs were more commonly recognized by community members than early-stage symptoms (13, 21). This pattern may be due to the highly visible nature of wounds and ulcers, which are often associated with severe disease stages commonly observed in affected communities. In contrast, early symptoms such as nodules, numbness, or patches may be overlooked because they are less visible, painless, or poorly understood. The implication is that many individuals may delay seeking healthcare until diseases progress to advanced stages, thereby increasing the risk of disability and prolonged transmission.

Sex differences in symptom recognition were also statistically significant, although awareness levels themselves did not differ significantly between males and females. Female respondents more frequently recognized symptoms such as skin lesions and patches, whereas males more commonly identified wounds. This finding aligns partly with the work of Ochola et al. (22), who observed that gender roles influence how diseases are perceived and managed within households and communities. Women, due to their caregiving responsibilities and closer involvement in family health matters, may be more attentive to subtle skin changes and early symptoms. Men, however, may focus more on visible and physically disabling manifestations that affect occupational functioning and physical labor. Despite these differences, the absence of major gender disparities in awareness suggests that public health messaging on skin NTDs may have reached both sexes relatively equally within the study communities.

The findings regarding perceived causes of skin NTDs are particularly important in understanding stigma and treatment-seeking behavior. Although bacterial infection and genetic factors were the most commonly identified causes overall, substantial proportions of respondents still attributed these diseases to supernatural causes and poor hygiene. This coexistence of biomedical and traditional beliefs has been documented in several previous studies in Ghana and other endemic countries (6, 11, 12). In rural and less educated populations, diseases affecting the skin are often interpreted through cultural and spiritual frameworks, particularly when symptoms become chronic or disfiguring. Such beliefs may contribute to delayed treatment-seeking, preference for traditional healers, and fear-driven stigma toward affected individuals.

The persistence of supernatural explanations in the present study also supports findings from Tuwor et al. (2), who documented experiences of stigma, isolation, and social exclusion among persons affected by Buruli ulcer and yaws in Ghana. Similarly, Dako-Gyeke et al. (9) reported that persons affected by leprosy in Ghana frequently experienced discrimination due to misconceptions about disease causation and transmission. These misconceptions may reinforce community fear and discourage open discussion of symptoms, thereby limiting early detection efforts. The findings, therefore, suggest that improving biomedical awareness alone may not be sufficient unless interventions also address deeply rooted cultural interpretations of disease.

The study further highlights the broader social consequences associated with skin NTDs. Misconceptions and fear surrounding these diseases contribute to stigma, social rejection, psychological distress, and reduced quality of life for affected individuals. These findings are consistent with studies from Bangladesh, Brazil, and Nepal, which demonstrated that persons affected by leprosy and related skin NTDs often experience anxiety, depression, social isolation, and reduced participation in community life (14, 16, 23). The persistence of stigma may also discourage healthcare utilization, particularly where visible deformities are associated with shame or spiritual punishment.

The findings of this study demonstrate that awareness of skin NTDs in Ghana is relatively widespread, but substantial gaps remain in accurate symptom recognition and understanding of disease causation. Educational attainment and geographical location significantly shaped awareness and perceptions, while gender differences were more evident in symptom interpretation than in overall awareness. The study, therefore, underscores the need for culturally sensitive interventions that combine biomedical education with community engagement strategies capable of addressing persistent myths and stigma. Public health campaigns should prioritize early symptom recognition, promote accurate information on disease causation, and involve community leaders, religious institutions, and persons with lived experiences in awareness creation. Strengthening community-based education and surveillance systems may further contribute to early detection, timely treatment-seeking, and reduced stigma associated with skin NTDs in Ghana.

### Strengths and limitations

A key strength of this study is the relatively large and diverse sample of 1,067 respondents drawn from four regions of Ghana, representing urban, peri-urban, and rural communities. This diversity enhances the breadth of perspectives captured and strengthens the applicability of the findings across different socio-demographic and geographical contexts. The use of a multi-stage sampling approach improved the representativeness of the sample, while the deployment of standardized electronic data collection tools through Kobo Collect helped ensure consistency, data quality, and reduced interviewer-related errors. Additionally, the assessment of multiple dimensions of community perceptions, including awareness, symptom recognition, and perceived causes of skin neglected tropical diseases (NTDs), provided a comprehensive understanding of public knowledge and beliefs. The application of chi-square statistical tests further enabled the identification of significant socio-demographic differences in awareness and perceptions across population groups. The study also contributes valuable insights by examining both biomedical and socio-cultural explanations of skin NTDs. By exploring perceptions related to bacterial infections, genetic predisposition, poor hygiene, and supernatural causes, the findings reveal the coexistence of scientific and traditional belief systems within communities. Understanding these diverse explanatory models is important for developing culturally appropriate health education strategies and interventions aimed at reducing misconceptions, stigma, and discrimination associated with skin NTDs.

Despite these strengths, certain limitations should be considered when interpreting the findings. The cross-sectional nature of the study limits the ability to establish causal relationships between socio-demographic characteristics and levels of awareness or perceptions of skin NTDs. The reliance on self-reported information may also have introduced recall bias and social desirability bias, as respondents may have provided answers they perceived to be socially acceptable rather than reflecting their actual beliefs or knowledge. Although the study included participants from four regions of Ghana, the exclusion of other regions and ecological zones may affect the extent to which the findings can be generalized to the entire country. Furthermore, the quantitative design primarily captured measurable patterns and associations but did not provide deeper insights into the underlying reasons, experiences, and contextual factors shaping perceptions of skin NTDs. The use of structured questionnaires may have further limited respondents’ ability to express complex or nuanced beliefs, potentially resulting in an incomplete representation of community understandings of these conditions.

## Conclusion

This study highlights that awareness, symptom recognition, and perceived causes of skin neglected tropical diseases in Ghana vary across socio-demographic and regional contexts. Although awareness was generally high, important gaps remain in recognizing early symptoms and understanding biomedical causes of these diseases. These findings underscore the need for targeted health education and community engagement strategies that address misconceptions, improve early recognition, and promote timely healthcare seeking. Strengthening integrated skin NTD control efforts through culturally appropriate interventions will be essential for reducing delays in diagnosis, stigma, and the ongoing burden of these conditions in Ghana.

### Recommendations

The Ghana Health Service, through the National Neglected Tropical Diseases Programme and regional/district health directorates, should strengthen targeted community education on skin NTDs in areas where low awareness, poor symptom recognition, and misconceptions were identified. Health education interventions should focus on improving recognition of early signs of leprosy, Buruli ulcer, and other skin NTDs, while addressing misconceptions related to supernatural causes, curses, and stigma. These interventions should be delivered through community health workers, local radio programs, schools, religious institutions, and traditional leaders using locally appropriate languages and messages.

District health authorities in endemic and underserved regions should integrate routine skin NTD screening into primary healthcare and community outreach activities. Community-based surveillance volunteers should be provided with regular training and resources to improve early identification, referral, and follow-up of suspected cases.

Healthcare providers should receive periodic training on skin NTD diagnosis, patient counseling, and stigma reduction to promote respectful and timely care for affected individuals. The Ghana Health Service should also strengthen collaboration with community leaders and persons affected by skin NTDs to develop stigma-reduction initiatives and promote social inclusion.

Future research should focus on evaluating the effectiveness of targeted awareness interventions and exploring regional differences in healthcare access, treatment-seeking behavior, and health system barriers to inform context-specific skin NTD control strategies.

## Data Availability

The raw data supporting the conclusions of this article will be made available by the authors, without undue reservation.
